# Visualization of the Glymphatic System Through Brain Magnetic Resonance in Human Subjects with Neurodegenerative Disorders: A Systematic Review and Meta-Analysis

**DOI:** 10.3390/jcm14124387

**Published:** 2025-06-19

**Authors:** Jana Hamzeh, Hayat Harati, Farah Ayoubi, Marie-belle Saab, Lea Saab, Elie Al Ahmar, Elias Estephan

**Affiliations:** 1 Neuroscience Research Center, Faculty of Medical Sciences, Lebanese University, Beirut P.O. Box 6573/14, Lebanon; janahamzeh@live.com (J.H.); hayat.harati@ul.edu.lb (H.H.); marie-belle.saab@prof.uls.edu.lb (M.-b.S.); 2 Department of Physical Therapy, Antonine University (UA), Baabda P.O. Box 40016, Lebanon; 3 Research and Innovation Department, Muscat University, Muscat 550, Oman; 4 Institut Universitaire de Kinésithérapie (IUK), Université Paris-Est Créteil Val de Marne—Université Paris 12, 94000 Paris, France; farah.ayoubi@gmail.com; 5 Laboratoire de la Motricité, Handicap et Rééducation (MOHAR), Lebanese University, Beirut P.O. Box 6573/14, Lebanon; 6 Faculty of Pedagogy, Lebanese University, Furn-El-Chebbak, Beirut P.O. Box 6573/14, Lebanon; 7 Faculty of Engineering, Sagesse University, Furn El Chebbak, Baabda P.O. Box 50501, Lebanon; lea.saab@uls.edu.lb; 8 LBN, University Montpellier, 34193 Montpellier, France

**Keywords:** MRI, glymphatic system, lymphatic drainage, neurodegeneration, brain, ALPS

## Abstract

**Background:** One of the major contributors to homeostasis at the level of the central nervous system, specifically the brain, is the glymphatic system, which is described as an exchange occurring at the level of and between the interstitial fluid and cerebrospinal fluid that has been linked to neurodegenerative processes. **Methods**: Fourteen studies were included after PROSPERO registration and a literature search. Screening, reviewing, and data extraction were performed by two reviewers. Quality assessment scales were used. General continuous and subgroup analysis, heterogeneity tests, and random effect models were run using SPSS. Forest plots were constructed based on subgroup analysis. **Results**: Significant correlations (*p* < 0.05) were detected between MRI indices and outcomes quantifying neurodegenerative diseases. Studies on Alzheimer’s disease showed a positive correlation between diffusivity indices and cognitive scores. Studies on Parkinson’s disease showed negative correlations between diffusivity indices and disease severity, progression, and motor function (*p* < 0.05). As for other conditions, the conclusions remain uncertain, yet positive results were detected (*p* < 0.05). **Conclusions**: Positive significant correlations were deduced between the ALPS index and cognitive scores, indicating that low cognition is correlated with a low ALPS index and enlarged PVSs. Negative significant correlations were deduced between ALPS indices and UPDRS scores, indicating motor dysfunction is correlated with lower ALPS indices and enlarged PVSs. Finally, MRI parameters may help to deduce disease progression across subgroups. Despite the presence of heterogeneity between studies, significant correlations with moderate to large effect sizes were detected. Glymphatic dysfunction measured through MRI indices is correlated with neurodegenerative changes across various neurological conditions.

## 1. Introduction

The blood–brain barrier, the “BBB”, has been previously hypothesized to be the major pathway through which oxygen and glucose are delivered to the brain. However, this mass transport, which is considered vital to brain metabolism, does not happen solely through the BBB. Indeed, the glymphatic system was first described in 2012 and has been drawing ample attention since then as it is thought to be one of the major contributors to homeostasis at the level of the central nervous system, specifically the brain [1,2]. The homeostatic phenomenon that governs the functioning of this system is known as glymphatic exchange, and it occurs at the level of and between the interstitial fluid (ISF) and cerebrospinal fluid (CSF) [3].

The process starts with the cerebrospinal fluid (CSF) flowing into the brain parenchyma through perivascular spaces (PVSs) at multiple levels and through association and projection fibers. The exchange takes place around several anatomical landmarks, including the rostral middle frontal PVS, basal ganglia, centrum semi-ovale, inferior frontal gyrus, parahippocampal gyrus, thalamus, and pons. This ISF–CSF interface is mediated by the Aquaporin-4 channels (AQP4), which are water channels spread throughout the brain and involved with several metabolic brain processes [3].

In fact, AQP4 is the most prevalent aquaporin in the CNS, with a major role in controlling the cerebral water balance. Recent studies have discussed the importance of AQP4 channels in CSF influx and lymphatic transport through linking AQP4 expression to several neurodegenerative processes. As a further indication of these channels’ importance, vascular end-feet create PVSs through which the CSF enters and spreads in the brain through the subarachnoid space. Then, arterial pulsatility carries the CSF into the brain parenchyma, facilitated by AQP4 channels in the astrocyte end-feet and around blood vessels. Finally, CSF mixes with the ISF in the extra-cellular space to exit the brain through the PVS along cranial and spinal nerves.

Based on this hypothesis, it may be deduced that the ISF does not only function as a cushioning entity for brain support and nourishment but may also be involved in mass transport, immunological function, and intercellular signal transmission [3]. In fact, several waste products and solutes are exported outside the central nervous system through meningeal and cervical lymphatic vessels [4,5].

Several in vitro and in vivo methods have been utilized in an attempt to visualize the glymphatic system. Two-photon microscopy has emerged as one of the first-choice methods to study glymphatic dynamics as it provides insights into the microscopic physiology of the glymphatic system. Furthermore, the introduction of this method allowed for the study of influx through perivascular space (PVS)–interstitial fluid (ISF) exchange and efflux through ISF drainage and was later translated into other imaging techniques. Tracer studies are among the candidate methods used. One study used In-DTPA as a tracer to visualize CSF flow throughout the brain [6]. Similarly, laser microscopy tracer studies using fluorescent agents have been applied to examine the glymphatic system, yet this method has some limitations, like it being an invasive method of quantification, in addition to providing limited input about subcortical regions [1].

Moreover, several studies have described using MRI for visualizing the glymphatic system and studying CSF dynamics. For instance, MRI coupled with intrathecal gadolinium-based contrast agents (GBCAs) has been used as a clinical tracer [7]. A study conducted in 2017 using MRI coupled with GBCA aimed to study glymphatic dynamics in patients with idiopathic normal pressure hydrocephalus (INPH). Enhancement was observed at different time frames overnight in the brain parenchyma, subarachnoid space, and intraventricular spaces at every time point in the sagittal sinus in both experimental and control groups, indicating sleep may contribute to glymphatic dynamics [8]. This suggests different gradients of clearance exist between different brain regions. Furthermore, dynamic contrast-enhanced MRI (DCE-MRI) has emerged as a viable method for the in-depth visualization of the glymphatic system through monitoring contrast dynamics in real time at the level of the AQP4 channels. Other MRI studies included T2-fluid-attenuated inversion recovery (FLAIR) and T1-weighted blood imaging, where enhancement was seen around the dural sinuses, meningeal arteries, and cribiform plate [9].

Additionally, phase contrast and diffusion tensor imaging (DTI) are frequently utilized in studying the glymphatic system in human subjects. The visualization of CSF dynamics using four-dimensional phase contrast MRI has been gaining attention, which may help in directly and non-invasively measuring human intracranial arterial hemo-dynamics [10], in addition to the visualization of CSF in spaces [11]. Alternatively, DTI involves administering a pair of motion probing gradients (MPGs) with an inverted 180° pulse between them. On DTI-MRI, tissues with strong diffusion are seen as low signal, while those of weak diffusion are seen as high signal, making it a promising method in CSF visualization [3]. One of the major advantages of DTI is its potential in the real-time monitoring of the studied system as time series through “diffusion tensor image analysis along the perivascular space” (DTI-ALPS) or (ALPS index). The ALPS score may be obtained through calculating a ratio of two sets of diffusivity values, indicating the influence of water diffusion along the perivascular space, which reflects the activity of the glymphatic systems [12].

Hence, the glymphatic system may be a pathway to understanding the pathophysiological processes involved in neurodegenerative disorders. In fact, numerous studies have highlighted the effect of sleep deprivation on glymphatic functioning in healthy individuals. These studies have shown that altered sleep patterns lead to Amyloid-beta (Aβ) accumulation in brain tissue, which may directly contribute to several disorders such as Alzheimer’s disease (AD) [13]. Moreover, excessive extra-axial fluid within six months of onset, which did not resolve after twenty-four months, was detected in patients with autism spectrum disorders [14]. Glymphatic dysfunction has also been reported in vascular disorders such as stroke and cerebral small-vessel diseases. As for strokes, whether ischemic or hemorrhagic in nature, impaired perfusion has been detected on MRI coupled with GBCA [15]. Finally, several studies examined glymphatic impairment among patients with movement disorders like Parkinson’s disease (PD), where negative correlations have been reported between ALPS indices and several outcome measures [3].

Hence, the optimal choice of the imaging techniques best suited to properly visualize the glymphatic system remains inconclusive. Additionally, the hypotheses of the glymphatic system entirely and the possible correlations between neurodegeneration and glymphatic dynamics remain uncertain and vague, which are the major reasons behind conducting this study. This is the first systematic review and meta-analysis with an aim to review and synthesize the data on glymphatic system dynamics and visualization methods using MRI across neurodegenerative disorders in humans, while assessing the risk of bias and quality of the included studies and discovering interactions between the studied variables. The resultant findings may aid in understanding the differences in glymphatic dynamics across various neurodegenerative diseases while comparing different quantification methods to identify the ideal ones. Furthermore, this study intends to provide a comprehensive and accurate synthesis of the available evidence on quantitative estimates obtained through MRI techniques, to objectively quantify the glymphatic functioning across the studied conditions.

## 2. Methods

### 2.1. Literature Search and Data Extraction

The literature search included studies that investigated the visualization and dynamics of the glymphatic system in neurodegenerative disorders of the brain. A systematic literature screening was conducted using three international databases, which were MEDLINE, PUBMED, and EMBASE. The following search strategy was utilized for data collection: (Glymphatic) OR (Brain) OR (Lymphatic drainage) AND (MR) OR (Magnetic Resonance) OR (MRI) OR (Neurodegeneration) OR (Healthy). The search included original studies up to 21 April 2024. Furthermore, a manual search was also performed by a blinded researcher, through screening the bibliographies of studies within the scope of the search to extract eligible articles.

For the data extraction process, two independent blinded reviewers extracted the data for synthesis after reviewing the obtained studies. The studies underwent three thorough screening processes, where the opinion of a third reviewer was considered when needed. The first screening was based on the titles, abstract, publication dates, and journal. The second screening was based on diagonal reading of the content of the articles after making sure the articles were of IMRAD format and met the eligibility criteria. Finally, the third and final screening for eligibility was based on thorough reading of the paper.

The following data were extracted: (1) Study characteristics, including author/s names, year of publication, enrollment period, study design, total number of participants, loss to follow-up, mean participant age, male-to-female ratio, controlling for covariates, ethical approval, IRB number, study protocol, outcome measures, tools used for analysis, the eligibility criteria, the statistical analysis used, and data reporting methods. (2) The utilized MRI technique, the MRI sequence used for assessing glymphatic dynamics, contrast used in case it was used, indices and ratios calculated, software used to generate the data, MRI scanning parameters, acquisition time points were applicable, analytical method used in glymphatic system assessment, targeted anatomical landmarks for data acquisition and measurement. As for the anatomical landmarks, major landmarks included the CSF space, brain parenchyma, basal ganglia, white matter volume, centrum semi-ovale, and perivascular spaces in the frontal lobe.

### 2.2. Inclusion Criteria 

**Figure 1:**
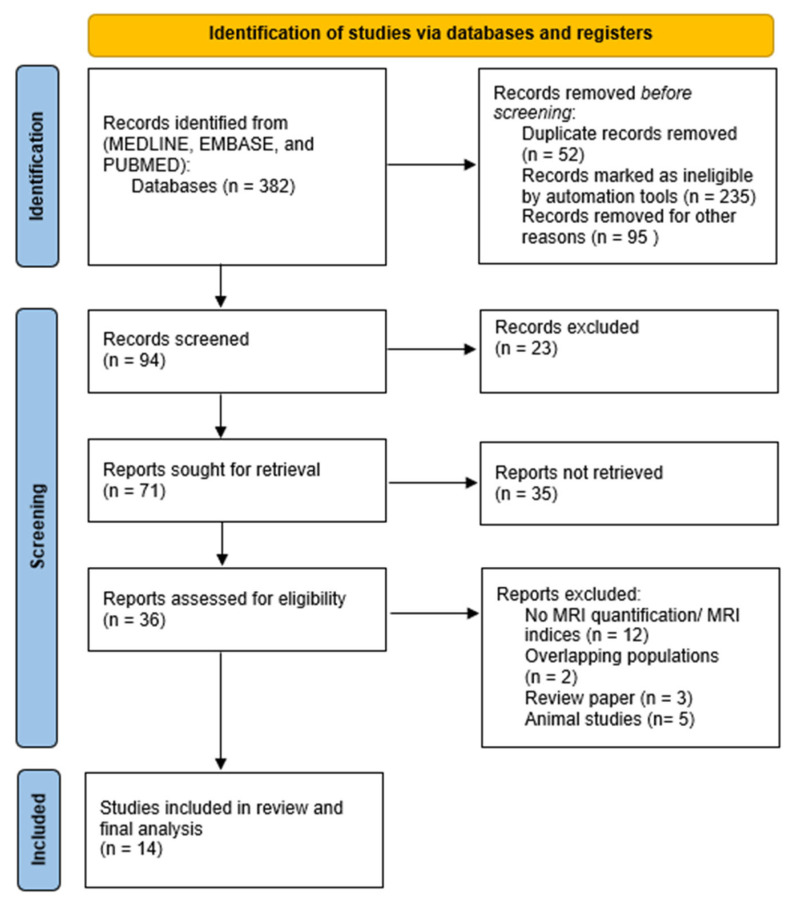
PRISMA flowchart of the study selection process (*n* = 14).

Following PROSPERO registration under the license CRD42024553673, the study selection was based on PRISMA guidelines and checklist, and the search process is illustrated in the PRISMA flowchart in Figure 1. Studies were eligible for inclusion if they met the following criteria: (1) Studies involved glymphatic system visualization and association with neurodegenerative disorders. (2) Studies reported MRI indices and ratios related to glymphatic dynamics. (3) Studies involved glymphatic system visualization and association with risk factors. (4) Studies involved glymphatic system visualization and neuroimaging signs of neurodegeneration. (5) Studies involved comparison of glymphatic dynamics between populations with neurodegenerative disorders and healthy controls. (6) Prospective or retrospective cohorts on the topic of glymphatic visualization. (7) Correlational studies on the topic of glymphatic visualization. (8) Experimental trials on the topic of glymphatic dynamics in neurodegenerative disorders.

### 2.3. Exclusion Criteria

Studies were excluded when the following criteria were detected: (1) Study was a case report. (2) Study included animal participants. (3) Studies were reviews without original data. (4) Studies included fewer than ten subjects. (4) Studies which were not published originally in English language. (5) Studies lacked MRI. (6) Studies did not provide any quantitative data.

### 2.4. Assessment of Risk of Bias and Quality

Two reviewers independently assessed the final included studies for risk of bias and quality using two standardized tools. “Newcastle-Ottawa Quality Assessment Scale”, a tool utilized to assess the quality of cross-sectional and case–control studies, in addition to non-randomized trials, was used to assess risk of bias through appraising study design. Additionally, “Quality Assessment of Studies of Diagnostic Accuracy (QUADAS)” tool was used to evaluate the quality of diagnostic studies that will be included in the final systematic review and meta-analysis.

### 2.5. Data Synthesis

IBM SPSS version 21 was employed for statistical analysis. First, quantification of quality assessment through continuous analysis was performed through calculating the mean of QUADAS scores. Then, continuous analyses were performed on the extracted data through descriptive analyses and subgroup analyses. Furthermore, and to quantify the observed correlations concerning the dynamics of the glymphatic system and relationship with neurodegeneration, Pearson correlation was performed after checking for assumptions to assess the (r) of correlation coefficient to deduce the correlation between the articles and designated variables. In terms of the major meta-analysis section, the conducted statistical tests included heterogeneity tests, effect size calculation, forest plot construction, in addition to random and fixed effects model. Finally, in terms of specific analyses targeted from these tests, the major correlations were between the following variables: ALPS index and outcome measures, diffusivity measures and outcome measures, MRI parameters and disease progression. *p* value was set to 0.05.

## 3. Results

### 3.1. Literature Search

We identified 382 studies in the initial systematic search of the selected databases. After the initial screening, 94 studies were left as eligible articles for further screening. Furthermore, after diagonal reading for the cardinal study characteristics of the 94 studies, only 36 were eligible for further analysis. Finally, one last thorough screening was performed by the blinded reviewers to evaluate the remaining studies. Only 14 articles were eligible to be included in the final synthesis and meta-analysis. The excluded articles were disregarded for the following reasons: (1) Three of the articles were review papers. (2) Five were based on animal studies. (3) A major number of the excluded articles included overlapping populations and did not control for covariates. (4) Some of the studies did not focus on methods of visualization and quantification of the glymphatic system in neurodegenerative disorders. (5) Some of the studies provided only qualitative analysis or invalid quantitative analysis.

### 3.2. Synthesis of Included Studies

Data synthesis included a descriptive analysis of the included studies, describing their characteristics, MRI sequences, and outcome measures used, in addition to an inferential analysis that included the quantitative synthesis of quality assessment and risk of bias in the final included studies. Additionally, continuous analysis using descriptive statistics was performed followed by random effects model analysis, forest plots based on the disease studied, and subgroup analysis based on MRI indices and glymphatic dynamics to deduce the correlation between the findings and neurodegeneration.

#### 3.2.1. Characteristics of the Included Studies

The total number of participants, split between patients with neurodegenerative conditions and healthy controls, was 3871. The number of participants varied widely between the studies as it ranged between a minimum of 30 participants and a maximum of 2452 participants. Most of the participants were elderly, yet most included studies controlled for covariates including age and gender. Included populations were patients diagnosed with cognitive impairments (mild and severe), patients diagnosed with Alzheimer’s disease, patients diagnosed with Parkinson’s disease (with/without freezing of gait and with/without cognitive impairment), patients diagnosed with cerebro-vascular disease (stroke leading to neurodegeneration), patients diagnosed with presbycusis (bilateral age-related hearing loss), and patients with small-vessel disease (infarcts leading to neurodegeneration). The eligibility criteria varied widely between studies, since the studies included participants diagnosed with several neurodegenerative disorders. As for the study types, the final included studies were either cross-sectional, case–control, or cohort studies, and the publication dates ranged between 2017 and 2024. The study characteristics are outlined in Table 1.

#### 3.2.2. MRI Sequence for Glymphatic Assessment

Most of the included studies utilized DTI as the sequence to study glymphatic dynamics across the different neurodegenerative disorders. One study used positron emission tomography (PET-MRI) alongside DTI in addition to 3D T1 MRI [18]. One study used FMRI with ECHO planar sequence [27], and one study used FLAIR sequence (3D T1 weighted) [29]. Most of the studies used a 3T MRI system for image acquisition, except for one study, which acquired the images using a 7T MRI system [26]. Moreover, for the studies that used DTI, the b-value, which is the factor that reflects both the strength and timing of gradients, ranged between b = (1000–3000 s/m^2^). As for the sequences employed, three studies acquired the images using a T2-weighted sequence (enhanced water signal) [21,24,26], while the remaining 11 used T1-weighted signal (suppressed water signal). As for other MRI parameters, Table 1 outlines details on the parameters used in an expanded manner. Additionally, two studies assessed CSF samples, and one study assessed dopamine transporter imaging in addition to the CSF samples. Moreover, one study assessed plasma readings, another assessed brain volumes through MRI, one assessed fLudeoxyglucose-18 (FDG) through PET, and one assessed brain temperature. The MRI characteristics are outlined in Table 1.

#### 3.2.3. Outcome Measures Used to Assess Clinical Characteristics

The studies that included patients with AD and cognitive impairments mainly used two valid quantitative outcome measures, which were mini mental state exam (MMSE) and Montreal cognitive assessment test (MoCA), to assess cognitive decline and reserve under different domains. The study on persbycusis used MoCA as an outcome measure to assess cognition in relation to age-related hearing loss [19]. The study on CVD used MoCA in addition to the Pittsburgh sleep quality index (PSQI) to assess the relationship between regression, degeneration, and sleep [25]. Finally, as for the studies that focused on PD, the majority used the unified Parkinson’s disease rating scale (UPDRS) to assess motor function, coordination ability, and functional ability in addition to MMSE and/or MoCA. The other utilized outcome measures are outlined in Table 1.

### 3.3. Assessment of Study Quality

**Figure 2:**
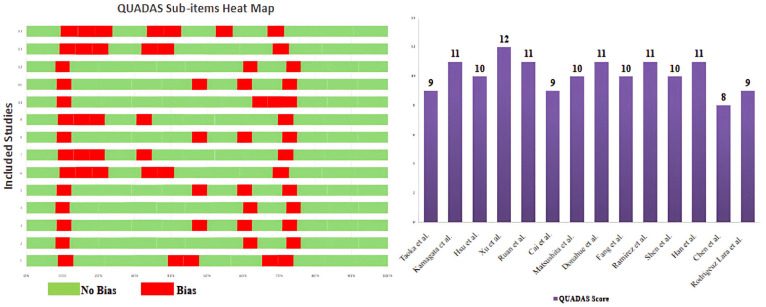
Heatmap graph and overall score of QUADAS of the included studies (*n* = 14) [12,16,17,18,19,20,21,22,23,24,25,26,27,28].

All 14 included studies showed low to moderate risk of bias in terms of patient selection, index test, reference standard and flow and timing, as per the QUADAS test (Figure 2), and in terms of selection, comparability, and exposure, as per the NOS tool for cross-sectional and case–control studies (Table 2). The major weak points of the studies were in terms of the eligibility criteria elaboration, image analysis explanation, characteristics of the control groups, in addition to baseline assessment and independence of observation measures.

### 3.4. Continuous Analysis Outlining Descriptive Statistics of Included Studies

**Figure 3:**
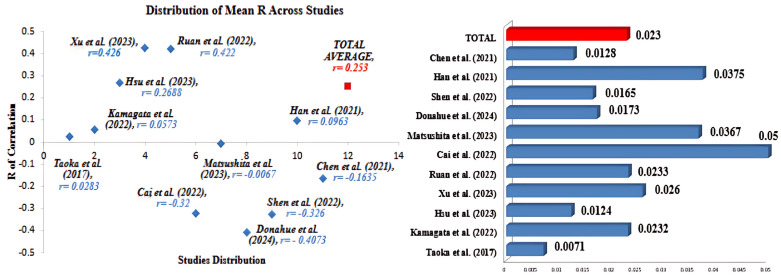
Distribution of r and *p*-values across included studies, where *p*-values are demonstrated in the bar chart to the left (*n* = 11) [12,16,17,18,19,20,21,22,25,26,27].

Eleven studies were included in the continuous analysis; the overall significance in terms of the *p*-value was detected between all studies, as shown in Figure 3, with *p* = 0.023 for the mean r of correlation, indicating the presence of significant correlation between neurodegeneration and glymphatic dysfunction. For studies that reported the r of correlation and included patients with AD, all the included studies reported a significant *p*-value, indicating the presence of a correlation between AD and glymphatic dysfunction. The general subgroup analysis resulted in an average of r = 0.0175 and *p* = 0.02, indicating a significant positive correlation. As for studies that included patients with PD, all included studies reporting r of correlation reported a significant *p*-value, indicating a relationship is present between PD symptoms and glymphatic impairment. The general subgroup analysis resulted in an average of r = −0.117 and *p* = 0.01928, indicating a significant negative correlation. Finally, studies that examined other conditions found a significant positive relationship existed between glymphatic dynamics and neurodegeneration, with r = 0.656 and *p* = 0.024.

### 3.5. Subgroup Analysis Using MRI Indices and Parameters’ Correlation with Outcome Measures

#### 3.5.1. ALPS Index and Outcome Measures

A significant positive correlation exists between the ALPS index and cognitive score in patients with AD measured through MMSE and MoCA primarily, where r = 0.414 and *p* = 0.02. As for patients with PD, a significant negative correlation was reported between ALPS index and UPDRS scores as outcome measures for motor function, planning, in addition to functionality and MMSE which was used to assess cognition, where r = −0.2 and *p* = 0.017. As for other conditions, a significant positive correlation was detected between ALPS index and outcome measures, mainly using MoCA, where r = 0.426 and *p* = 0.026. In fact, it may be deduced that diffusivity along the glymphatic system is affected in patients with cognitive impairment such as AD and as a cardinal feature of neurodegeneration. Therefore, decreased diffusivity is among the altered glymphatic dynamics associated with neurological diseases, as measured through the ALPS index. The ALPS index may be considered a reliable measure in quantifying cognitive decline and motor function decline. Based on the latter, the findings are demonstrated in Table 3.

#### 3.5.2. PVS and Outcome Measures

A significant negative correlation exists between PVS and cognitive scores in patients with AD, measured through MMSE and MoCA primarily, where r = −0.327 and *p* = 0.01. As for patients with PD, a significant negative correlation was reported between PVS index and UPDRS scores as outcome measures for motor function, planning, functionality and MMSE, where r = −0.02 and *p* = 0.02. As for other conditions, a significant positive correlation was detected between PVS index and outcome measures, mainly using MoCA, where r = 0.775 and *p* = 0.023. Therefore, it may be deduced that enlarged PVS is another major finding associated with neurodegeneration. Enlargement of the PVS in the brain signifies altered glymphatic dynamics through altering the CSF-ISF exchange and is associated with cognitive decline, aging, small-vessel disease, and motor function decline. Hence, PVS calculations using DTI are another reliable measure of glymphatic function in neurological conditions. The findings are demonstrated in Table 3.

#### 3.5.3. MRI Parameters and Disease Progression

Major MRI parameters used in this analysis are the TR, TE, acquisition method, flip angle, degree of diffusion weighting (b), and sequence used. The results outline a very significant positive correlation between MRI parameters and disease progression in patients with AD, with r = 0.22 and *p* = 0.008. Additionally, the results outline a very significant negative correlation between MRI parameters and disease progression, with r = −0.42 and *p* = 0.0003. As for all included studies, a negative correlation existed between MRI parameters and disease progression, with r = −0.097 and *p* = 0.004. The most variable parameter behind the significance may be the degree of diffusion weighting. As (b) increases, sensitivity to diffusion increases. This is consistent with the influx and efflux dynamics of the glymphatic system, which may become impaired with neurodegenerative processes. The findings are demonstrated in Table 3.

### 3.6. Random Effects Model Across Subgroups

Based on the findings presented in Table 4, for the ALPS index and outcome measures, irrespective of the condition studied, a small effect size of d = 0.22 was detected, yet the correlation between ALPS and outcome measures in the random effects model was of high significance (*p* = 0.002) with medium heterogeneity, I^2^ = 45% and Tau^2^ = 0.02, indicating low variability. Furthermore, for PVS and outcome measures, a small effect size of d = 0.28 was detected, yet the correlation between PVS and outcome measures in the fixed effect model was of high significance (*p* = 0.0008) with medium heterogeneity, I^2^ = 50% and Tau^2^ = 0.025, indicating low variability. Finally, as for MRI parameters and disease progression, only three studies reported correlations on this matter, yet the effect size is very high, with d = 1.4. Additionally, the correlation between MRI parameters and disease progression was of high statistical significance (*p* = 0.007) with high heterogeneity, I^2^ = 60% and Tau^2^ = 0.06, indicating low variability. The resultant heterogeneity may be secondary to several factors, such as within- and between-subject differences, different MRI parameters used, in addition to different stages of neurodegeneration and possible treatments being administered.

### 3.7. Forest Plot Analyses Based on Pathology

**Figure 4:**
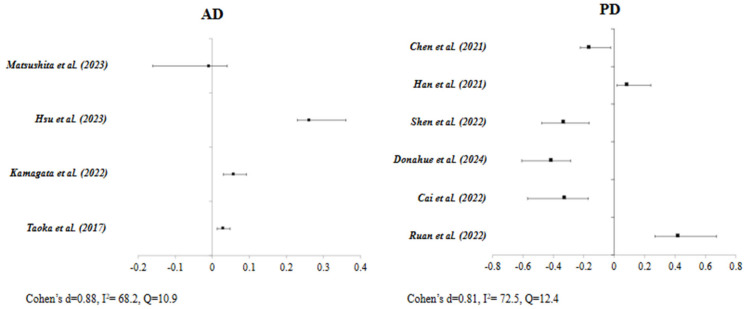
Forest plot of Pearson’s coefficient of studies based on pathology type [12,16,17,19,20,21,22,25,26,27].

The forest plot in Figure 4, for articles studying the relationship between AD in terms of cognition, disease progression and glymphatic dysfunction, shows that most of the studies are skewed to the right while reporting a positive correlation between AD progression and cognitive decline with MRI indices, specifically the ALPS index. In other words, the higher the ALPS index, the better the cognitive function, and vice versa, with a big effect size d = 0.88 and high heterogeneity across the included studies I^2^ = 68.2.

As for the forest plot for articles studying the relationship between PD in terms of motor function, coordination, and function assessed through UPDRS and cognition assessed through MMSE and MoCA, as shown in Figure 4, we may deduce that the majority of the studies are skewed to the left while reporting a negative correlation between UPDRS and PD outcomes and ALPS and diffusivity mainly. Hence, we may deduce that disease regression and progressiveness quantified through a higher UPDRS score are associated with decreased ALPS index and impaired PVS, with a big effect size d = 0.81 and high heterogeneity I^2^ = 72.

## 4. Discussion

This systematic review, and meta-analysis, is the first to review the available literature on current MRI-based studies aiming to visualize and quantify glymphatic dynamics in neurodegenerative populations.

The included studies focused on evaluating MRI indices, such as the ALPS index, PVS changes, WMH, and GMV, mainly using DTI-MRI. Furthermore, the outcome measures used to quantify the included neurodegenerative disease were majorly MMSE and MoCA for cognitive function quantification in AD and PD patients; this is consistent with past studies on neurological diseases where these scales were used and considered to be reliable and valid for measuring cognitive function in these populations, with similar scores. Yet, these scores have some limitations, such as the inability to be translated properly across different cultures [29]. Additionally, UPDRS was used for the quantification of motor function, coordination, overall function, disease severity, and progression in PD patients similarly to past studies showing similar scores. Despite UPDRS being a widely used, valid and reliable tool, among its major limitations is its subjectivity [30].

Evident positive correlation was present between ALPS index and PVS changes predominantly and cognitive score (MMSE and MoCA) in AD patients. In terms of pathophysiology, a lower ALPS index indicates neurodegeneration, neuroinflammation, and glymphatic dysfunction. Moreover, lower cognitive scores on MMSE and MoCA reveal lower cognitive reserve and decreased cognitive function. The latter explains the correlation found across all articles, with a large effect size and significant fixed and random effects analysis, despite heterogeneity. In fact, as the ALPS index decreases and PVS enlarges, glymphatic dynamics become impaired, which, in turn, leads to the accumulation of waste products and impaired ISF-CSF exchange. The latter would increase cognitive decline secondary to these altered processes and may, in turn, be affected by cognitive impairment. Causality between the two may not be concluded in this study and should be targeted in future ones.

As for studies on PD, significant negative correlations were detected between ALPS index and PVS changes and outcome measures (UPDRS) predominantly (*p* < 0.05). In fact, a higher UPDRS score indicates lower motor function, lower coordination, and lower overall function, which explains why it is inversely correlated with ALPS. Hence, lower diffusivity may be associated with motor dysfunction, in addition to enlarged or distorted PVS. This justifies the correlation found across articles on PD, where the negative correlation existed with large effect size estimates, despite the heterogeneity across the included studies. Finally, as for other conditions, results like those seen in AD and PD populations were detected (*p* < 0.05). Additionally, MRI parameters were reported to be correlated with disease progression in other conditions and PD despite heterogeneity, with a large effect size (*p* < 0.05). This may be explained by the decreased exchange between the CSF and ISF, which is crucial for brain homeostasis and decreases in waste drainage, leading to disease progression.

In fact, previously, it was thought that the CNS lacks a specific CSF-ISF exchange mechanism [31]. Yet, during the past decade, several studies suggested the opposite, and the collective results of these studies succeeded in showing the presence of specific anatomical pathways governing the process of flow and exchange between CSF and ISF at the level of the brain, known as glymphatic exchange [1,32].

Furthermore, several studies have attempted to visualize the glymphatic system and study its dynamics in humans using different MRI modalities and protocols [9,33]. The findings of the studies in terms of correlations between disease characteristics and MRI changes are similar to the findings reported in this study, specifically in terms of diffusivity and ALPS indices, in addition to correlation results. Studies mainly managed to show the existence of associations between glymphatic functioning and brain physiology, in addition to glymphatic functioning and neurodegeneration, across a vast range of disorders, including AD, PD, CVD, traumatic brain injuries, small-vessel disease, aging [34,35], and psychiatric disorders. Hence, it may be deduced that there are uniform findings between the observations reported in this study and previous studies on the topic, where similar correlations were detected across similar anatomical landmarks using similar MRI protocols [3].

Moreover, DTI-MRI in the included studies showed results similar to other studies in the literature, which utilized the same protocols, where the ALPS index and PVS proved to be reliable measures of the glymphatic system, specifically in the basal ganglia, frontal lobe, association and projection fibers in the parenchyma, and centrum semi-ovale [3].

Yet, the hypothesis about the glymphatic dynamics is still debatable, specifically considering neurodegenerative disorders that are different, progressive, and multi-factorial in nature. Therefore, reviewing and synthesizing quantitative data on this hypothesis and how they may be correlated with various disorders and changes may be a crucial step towards the establishment of a well-defined and measurable theory that can be reproduced and generalized. The major strength of this study is in it being the first meta-analysis of its kind aiming to review the available literature; praise the quality; test the heterogeneity, variance, effect size estimates, and fixed and random effects on the correlations between the glymphatic dynamics detected through reliable MRI indices and outcome measures quantifying neurodegenerative diseases. Additionally, this is the first study to utilize a methodology where not only general continuous analysis was performed but also subgroup analysis while running all meta-analysis-related tests on the subgroups to formulate precise conclusions and compare findings. Finally, this is the first study to utilize such strict inclusion criteria in this domain, which was proven successful despite the heterogeneity according to the results. The results presented in this review and meta-analysis may serve as a milestone for future studies to assist in discovering in-depth correlations between glymphatic functioning and neurodegeneration and support the uncertain hypothesis on this topic.

As for the limitations of this study, the major limitations may be due to the heterogeneity of the included studies, despite using strict and successful inclusion criteria, which may affect the generalizability; variance seen within and between the different included studies; the novelty of the area being studied, which yields a lower number of articles compared to other topics; the non-uniformity of methodologies between studies on the topic, which led to the exclusion of a big number of pooled studies; the absence of control groups in various studies in the literature; the incapacity or inappropriateness of statistical methods employed by some studies, which led to their exclusion; and the loss to follow-up in many studies, which also led to exclusion. Other limitations include the absence of studies performing follow-up at uniform time frames to understand disease progression and duration and insufficient covariate analyses in some studies.

Future studies on the topic are invited to construct uniform and well-thought-out methodologies for hypothesis testing and for translation into clinical practice as well, including feasibility studies and behavioral studies. This may be possible through controlling for more covariates that have a direct effect on within- and between-subject differences, utilizing different measurement protocols to compare the most objective ones, studying glymphatic dynamics across various populations to compare findings, and proposing follow-up regimes. Future research on the topic should include longitudinal data to assess the correlation between glymphatic function, disease progression, and duration. Furthermore, researchers should focus on performing multiple regression analysis to understand direct factors and predict changes in neurodegeneration based on the glymphatic system.

In fact, this study may serve as a basis for understanding changes in terms of glymphatic dynamics in neurodegenerative conditions in a better way while highlighting objective methods to quantify and measure these changes. The synthesis describes how changes may differ depending on the neurodegenerative processes and disease type while providing explanations for the differences. The findings may also assist in understanding the pathophysiological and physiological processes behind neurodegeneration and glymphatic function while proposing diagnostic methods and criteria. The latter would aid in developing multi-disciplinary treatment approaches from various medical backgrounds and professions to target neurodegenerative diseases through understanding the processes behind them.

## 5. Conclusions

The findings presented in this study may provide a foundation for understanding glymphatic dynamics in neurodegenerative diseases of the brain. Based on the findings and despite the presence of heterogeneity between the studies due to covariants and internal differences, significant correlations with moderate to large effect sizes were detected, not only across all studies in general continuous analysis but also across subgroup analysis. Summing up, positive significant correlations may be deduced between ALPS index and cognitive scores, indicating low cognition is correlated with low ALPS and enlarged PVS. Negative significant correlations may be deduced between ALPS and UPDRS scores, indicating motor dysfunction is correlated with lower ALPS and enlarged PVS. Finally, MRI parameters may explain disease progression across the subgroups. Yet, more robust methodologies aiming to link neurodegeneration with glymphatic dynamics are needed to decrease the observed variance and heterogeneity and to enable researchers to translate the findings into clinical practice.

## Figures and Tables

**Table 1:** Summary of findings of the articles included in final analysis (*n* = 14).

	Author	Date	Type of Study	Participants	Outcome Measures	MRI Parameters	Results
1	Taoka et al. [12]	2017	Cross-sectional	31 (AD, MCI, SCI)	DTI-MRI: FA, ALPS, MMSE	3T, b = (0, 1000, 2000 s/m^2^), TR = 6600 ms, TE = 89 ms, FOV= 230 mm, slice thickness = 3 mm	b = 1000: Diffusivity PVS/MMSE: **(r = 0.40, *p* = 0.026), (r = 0.50, *p* = 0.0042)**; Diffusivity Projection Fibers/MMSE: **(r = −0.52, *p* = 0.002)**; Diffusivity association fibers/MMSE: **(r = −0.55, *p* = 0.0013).** b = 2000: Diffusivity Projection fibers/MMSE: **(r = −0.48, *p* = 0.0067)**; ALPS/MMSE: **(r = 0.46, *p* = 0.0084).**
2	Kamagata et al. [16]	2022	Case-Control	31 HC, 44 MCI, 36 AD	DTI-MRI: PVS, ALPS, FW. MMSE, MOCA, FAQ, FDG/ADAS/ CDR-SB/Aβ42	DW, T1-weighted, FLAIR using 3T MRI	Higher PVS-BG/worse FAQ score **(r = 0.42, *p* = 0.026)** Higher FW-WM/lower CSF-Aβ42 **(r = −0.47, *p* = 0.021)**, worse MMSE **(r = −0.41, *p* = 0.021)**, worse FAQ **(r = 0.36, *p* = 0.044)** Lower ALPS/Lower CSF-Aβ42 **(r = 0.41, *p* = 0.026)**, FDG-PET SUVR **(r = 0.54, *p* < 0.001)**, worse MMSE **(r = 0.41, *p* = 0.026)**, FAQ **(r = −0.38, *p* = 0.016)**, CDR-SB **(r = −0.47, *p* = 0.003)**, ADAS-11 **(r = −0.40, *p* = 0.013).**
3	Hsu et al. [17]	2023	Cross-sectional	50 = 13 HC, 37 AD	PET-MRI: ALPS, FA, GMV. MMSE, CDR, CERAD-NAB	PET-MRI: TR = 6000 ms, TE = 392 ms, TI = 2100 ms. 3D T1: TR = 2000 ms, TE = 2.67 ms, T1 = 900 ms DTI = b = 1000 s/mm^2^. TR = 8800 ms, TE = 91 ms	ALPS/CERAS-NAB: **(R^2^ = 0.35, *p* < 0.01)**, MMSE **(R^2^ = 0.33, *p* < 0.01)**, and total GMV ratios **(R^2^ = 0.27, *p* < 0.01).**
4	Xu et al. [18]	2023	Cross-sectional	100, 60 with presbycusis	DTI-MRI: ALPS, MOCA	3T, TR = 10 s, TE = 95 ms, slice thickness = 2 mm, b value (0–1000 s/mm^2^)	ALPS/MOCA: **(rho = 0.426, *p* = 0.026)**
5	Ruan et al. [19]	2022	Case-control	59 PD with/without FOG, 34 HC	DTI-MRI: ALPS. FOG-Q, UPDRS, MMSE, GFQ	3T, TR = 8700 ms, TE = 102 ms, b (0–2000 s/mm^2^)	DTI-ALPS/illness duration **(r = 0.511, *p* = 0.003)**, UPDRS-III total **(r = 0.369, *p* = 0.038)** Decreased ALPS in PDFOG and PD-nFOG **(*p* < 0.05)**.
6	Cai et al. [20]	2022	Retrospective data	93 PD, 42 HC	DTI-MRI: ALPS. MMSE, UPDRS, Hamilton Anxiety and Depression scores, PDSS	T1: TR/TE = 580/18 ms T2: TR/TE = 5100/130 ms. FLAIR: TR/TE = 9600/110 ms DTI = TR/TE = 8000/76 ms, b (0–1000 s/mm^2^)	Lower ALPS in PD **(β = −0.143, *p* < 0.001)**, ALPS/UPDRSIII in PD patients of older age **(r = −0.315, *p* < 0.05)**, ALPS/disease duration in PD patients of older age **(r = −0.325, *p* < 0.05)**.
7	Matsushita et al. [21]	2023	Retrospective data	58 total, 29 with AD, 29 HCs	DTI-MRI: ALPS, SUVR, Diffusivity PVS. MMSE, brain temperature	T1-weighted: TR/TE = 2300/2.919 ms DTI: b (0–1000 s/mm^2^), TR/TE: 11,000/87 ms	ALPS/age: **(r = −0.43, *p* < 0.05)** in AD patients SUVR/MMSE: **(r = −0.027, *p* < 0.001)**
8	Donahue et al. [22]	2024	Cross-sectional	50 PD	MRI: PVS MDUPDRS, MOCA, neuropsychological assessment	3T, TR/TE/TI = 2400/2.22/1000 ms	Rostral middle frontal PVS volume/MOCA: **(r = −0.524, *p* < 0.001),** global cognition score: **(r = −0.380, *p* = 0.007)**, visuospatial function **(r = −0.49, *p* < 0.001)** Basal ganglia PVS/MOCA: **(r = −0.318, *p* = 0.029)**, global cognition score **(r = −0311, *p* = 0.029)**, and memory **(r = −0.308, *p* = 0.031)** Centrum semi-ovale PVS/MOCA: **(r = −0.358, *p* = 0.011)**, memory **(r = −0.374, *p* = 0.007)**
9	Fang et al. [23]	2020	Prospective data	287 PD and 129 HC, 42 PD and 31 HC excluded	MRI: PVS, MDUPDRS, MOCA, Dopamine transporter imaging, CSF samples	T2 1.5 T or 3T, axial acquisition total time of 5 and 8 min	Centrum semi ovale PVS/CSF α-synuclein **(estimate = 98.6, 95% CI (3.1–194), *p* < 0.05)** Basal Ganglia PVS/CSF α-synuclein **(estimate = 138.2, 95%CI (6.7, 269.7), *p* < 0.05)** Centrum semi ovale PVS/CSF t-tau: **(estimate = 10.5, 95% CI (1.5–19.5), *p* = 0.023)** Basal Ganglia PVS/CSF t-tau: **(estimate = 12.8, 95% CI (0.4–25.5), *p* = 0.045)**
10	Ramirez et al. [24]	2021	Cross-sectional	*n* = 152 (CVD)	MRI: PVS, MOCA, PSQI	NA	PVS/PSQI: **(r = 0.70, *p* = 0.04)** BG PVS higher/Daytime Dysfunction higher: **OR = 5.31, 95% CI: 1.38–22.26), *p* = 0.018)**
11	Shen et al. [25]	2022	Cross-sectional	76 PD, 48 HC	DTI-MRI: ALPS, PVS, WMH. MMSE, UPDRS, Hamilton Anxiety and Depression scores	7T, TR = 5000 ms, T_1_1/T_1_2 = 900/2750 ms, TE = 2.3 ms. T2: TR = 7000 ms, TE = 66 ms DTI = TR = 6000 ms, TE = 71.8 ms, b (0–3000 s/mm^2^)	ALPS/UPDRSIII **(r = −0.24, *p* = 0.04)**, and UPRDS total **(r = −0.27, *p* = 0.02)** ALPS/disease duration: **(r = −0.30, *p* = 0.01)** PVS/MMSE: **(r = −0.4, *p* < 0.01)** in basal ganglia. ALPS/PVS in R hemisphere: **(r = −0.42, *p* = 0.0003).**
12	Han et al. [26]	2021	Cross-sectional	60 PD, 58 HC	FMRI: gBOLD and CSF flow, MOCA, UPDRS	ECHO flip angle = 90°, spatial resolution = 3 × 3 × 4 mm^3^, slice thickness = 3 mm, TR/TE = 2000/30 ms	Mean gBOLD correlation with positive peak at –4 s **(r = 0.28, *p* < 0.0001)** and negative peak at +4 s **(r = 0.37, *p* < 0.0001)** gBOLD/CSF correlation with MOCA **(r = −0.36, *p* = 0.012)**
13	Chen et al. [27]	2021	Prospective data	88 PD, into groups PD without cognitive impairment, with mild cognitive impairment, and with dementia	MRI: ALPS, UPDRS, MMSE, Plasma readings	3T, TR/TE: 15,800/77 ms, b = 1000 s/mm^2^.	PD-MCI and PD-D groups showed lower ALPS compared to PD wthout cognitive impairment **(*p* = 0.012, *p* < 0.001).** ALPS/MMSE: **(r = 0.222, *p* = 0.013)** ALPS/UPDRS total: **(r = −0.307, *p* = 0.005)**
14	Rodrigeuz Lara et al. [28]	2023	Prospective data	2452 scans of patients with small vessel disease	MRI: PVS, brain volumes	3D T1-weighted (2–3 FLAIR)	Inverse relationship between **PVS burden** and **total brain volumes**, inverse relationships between **PVS burden** and **gray matter volume (*p* < 0.05)** Higher **odds of covert brain infarcts in grade III PVS compared to grade I in basal ganglia (OR = 5.27, 95% CI = 3.01, 9.22) and centrum semi ovale (OR = 1.99, 95%CI = (1.16, 3.43)**

Bold text indicates major findings of the included studies. Abbreviations: AD: Alzheimer’s disease, MCI: Mild Cognitive Impairment, SCI: Subjective Cognitive Impairment, DTI: Diffusion Tensor Imaging, MRI: Magnetic Resonance Imaging, FA: fractional ansiotropy, ALPS: Index of Diffusivity along peri-vascular space (whenever speaking about diffusivity), MMSE: mini mental state exam, T: tesla, TR: Repetition time, TE: Echo time, FOV: Field of vision, PVS: perivascular space, FW: fractional volume of free water, MOCA: Montreal cognitive assessment test, FAQ: Functional activities questionnaire, FDG: flurodeoxyglucose, ADAS: Alzheimer’s Disease Assessment Scale, CDR-SB: Clinical Dementia Rating-Sum of boxes, FLAIR: Fluid attenuated inversion recovery, PVS-BG: Perivascular space in Basal Ganglia, FW-WM: functional volume of free water in the white matter, CSF: cerebrospinal fluid, PET: Positron Emission Tomography, SUVR: Standardized uptake value ratio, HC: healthy controls, GMV: Gray matter volume, CERAD-NAB: Consortium to Establish a Registry for Alzheimer’s Disease, TI: Inversion time, FOG: Freezing of gait, UPDRS: Unified Parkinson’s Disease Rating Scale, GFQ: General functioning questionnaire, FOG-Q: Freezing of gait questionnaire, NEX: number of excitations, PDSS: Panic disorder severity scale, PSQI: Pittsburgh sleep quality index, WMH: White matter hyper-intensities, PD: Parkinson’s Disease, PD-MCI: Parkinson’s Disease with mild cognitive impairment, PDD: Parkinson’s Disease with dementia, CVD: Cerebro-vascular Disease.

**Table 2:** Newcastle Ottawa Scale (NOS) of the included articles (*n* = 14).

Article	Selection				Comparability	Exposure		
	1	2	3	4	1	1	2	3
Taoka et al. [12]	✬					✬	✬	
Kamagata et al. [16]	✬					✬	✬	
Hsu et al. [17]	✬		✬		✬✬	✬	✬	
Xu et al. [18]	✬		✬		✬✬	✬	✬	
Ruan et al. [19]	✬		✬		✬✬	✬	✬	
Cai et al. [20]	✬		✬		✬✬	✬	✬	
Matsushita et al. [21]	✬		✬		✬✬	✬	✬	
Donahue et al. [22]	✬		✬		✬✬	✬	✬	
Fang et al. [23]	✬		✬		✬✬	✬	✬	
Ramirez et al. [24]	✬		✬		✬✬	✬	✬	
Shen et al. [25]	✬		✬		✬✬	✬	✬	
Han et al. [26]	✬		✬		✬✬	✬	✬	
Chen et al. [27]	✬		✬		✬✬	✬	✬	
Rodriguez Lara et al. [28]	✬		✬		✬✬	✬	✬	

✬: one point allocation, ✬✬: two points allocation.

**Table 3:** Subgroup analysis based on indices and MRI parameters (*n* = 11).

Subgroup	ALPS/Outcome Measures	*p*	PVS/Outcome Measures	*p*	MRI/Disease Progression	*p*
AD	0.373	0.02	−0.327	0.018	0.22575	0.008
PD	−0.2	0.017	−0.02	0.02	−0.42	0.0003
Other	0.426	0.026	0.775	0.023	-	-
All	0.213	0.021	0.0966	0.018	−0.097	0.004

**Table 4:** Random effects model for subgroups based on indices.

Random Effect Model							
	Effect Size	Variance	CI (95%)	*p*	Q	I^2^	Tau^2^
ALPS/outcome measures	0.22	0.02	[0.1–0.34]	0.002	12.34	45%	0.02
PVS/outcome measures	0.28	0.02	[0.16–0.40]	0.0008	15.67	50%	0.025
MRI/Disease Progression	1.4	0.55	[0.4–2.4]	0.007	20.5	60%	0.06

## Data Availability

The authors confirm that the data supporting the findings of this study are available within the article. Statistical analysis and protocols that support the findings of this study are available on request from the corresponding author.
